# Analysis of Ca^2+^-mediated sperm motility to evaluate the functional normality of the sperm-specific Ca^2+^ channel, CatSper

**DOI:** 10.3389/fcell.2024.1284988

**Published:** 2024-02-07

**Authors:** Jae Yeon Hwang

**Affiliations:** ^1^ Department of Molecular Biology, Pusan National University, Busan, Republic of Korea; ^2^ Institute of Systems Biology, Pusan National University, Busan, Republic of Korea

**Keywords:** sperm motility, hyperactivation, CatSper, Ca^2+^, computer-assisted semen analysis

## Abstract

Ca^2+^ is a key secondary messenger that modulates sperm motility by tuning flagellar movement in various species. The sperm-specific Ca^2+^ channel, CatSper, is a primary Ca^2+^ gate that is essential for male fertility in mammals. CatSper-mediated Ca^2+^ signaling enables sperm to develop hyperactivated motility and fertilize the eggs in the female tract. Therefore, altered CatSper function compromises the entry of Ca^2+^ into the sperm, followed by impairing hyperactivation and male fertility. However, methods to evaluate the function of the CatSper channel are limited to patch clamping and functional imaging using Ca^2+^ dye. Previous studies have revealed that various parameters for sperm motility are highly correlated with intracellular Ca^2+^ levels in mouse. Here, I cover a step-by-step protocol to analyze the change in Ca^2+^-mediated sperm motility by using computer-assisted semen analysis (CASA) to evaluate the functional normality of the CatSper channel in sperm. This approach analyzes sperm motility parameters during intracellular Ca^2+^ chelation followed by *in vitro* capacitation to recover intracellular Ca^2+^ via the activated CatSper channel. Thus, this Ca^2+^-handling method is handy and could be broadly applied in reproductive biology labs and clinics that have CASA equipment to examine the functional normality of the CatSper channel.

## Introduction

Sperm from various species apply Ca^2+^-mediated signaling pathways to modulate their flagellar movement and motility pattern. In marine invertebrates, such as sea urchins, the sperm acquire chemotactic movement by coordinating Ca^2+^ entry and downstream signaling pathways (Wood et al., 2005; Seifert et al., 2015). Mammalian sperm develop a unique motility pattern called hyperactivated motility via extracellular Ca^2+^ influx in the female reproductive tract (Suarez et al., 1993). The changed motility patterns enable the sperm to successfully migrate to eggs followed by fertilization, highlighting that Ca^2+^-mediated motility change is crucial for sperm fertility.

In the female reproductive tract, mammalian sperm acquire fertilizing ability in a process called capacitation, which triggers the sperm to develop hyperactivated motility by introducing extracellular Ca^2+^ (Yanagimachi, 1970; Suarez et al., 1993). The major pathway for Ca^2+^ entry in mammalian sperm is the sperm-specific ion channel, known as CatSper, on their flagella (Ren et al., 2001). The CatSper channel is a multi-protein complex comprising at least 14 proteins (Hwang and Chung, 2023). Previous studies using genetically engineered mouse models revealed that CatSper deficiency (Ren et al., 2001; Quill et al., 2003; Qi et al., 2007; Chung et al., 2011) or its altered function (Chung et al., 2017; Hwang et al., 2019) causes male infertility or subfertility due to the impaired sperm hyperactivation. In addition, mutations in the genes encoding CatSper subunits, such as CatSper1 (Avenarius et al., 2009), CatSper2 (Avidan et al., 2003; Smith et al., 2013; Luo et al., 2019; Schiffer et al., 2020), and CatSperε (Brown et al., 2018), have been identified in infertile males whose sperm failed to develop hyperactivated motility (Schiffer et al., 2020; Young et al., 2023). Thus, examining CatSper function is important to explain the physiological defects in sperm hyperactivation and male infertility. Yet, despite its significance, evaluating CatSper function is limited to patch-clamping techniques (Kirichok et al., 2006; Lishko et al., 2011; Strunker et al., 2011) and functional imaging using Ca^2+^ dye.

A previous study revealed that the intracellular Ca^2+^ level ([Ca^2+^]_i_) in sperm is correlated with male fertility (Kelly et al., 2018). Sperm from subfertile patients showed reduced sensitivity against progesterone (Kelly et al., 2018), which activates the CatSper channel in human sperm (Lishko et al., 2011; Strunker et al., 2011); this resulted in a significantly reduced [Ca^2+^]_i_ and Ca^2+^ oscillation. In addition, another study revealed that flagellar movement can be switched by the threshold [Ca^2+^]_i_ in the sperm (Sanchez-Cardenas et al., 2018). These studies suggest that [Ca^2+^]_i_ determined by CatSper function could be highly correlated with sperm motility patterns.

Here, I provide another detailed method and protocol—a sperm Ca^2+^-handling assay—which evaluates CatSper function by analyzing the [Ca^2+^]_i_-dependent motility change of mouse sperm, as described previously (Hwang et al., 2019; Hwang et al., 2022). This technique uses a computer-assisted semen analysis (CASA) system, instead of equipment for patch clamping and/or functional imaging. Thus, this protocol could be broadly used in andrology and reproductive biology labs that have a CASA system to evaluate CatSper function in mammalian sperm.

## Materials and equipment

### Materials and reagents

• Mice—all the mouse lines used in this study are on a C57BL/6 background.

- Wildtype (WT) mice (Charles River Laboratory)
- Mice of your interest
- CatSper-deficient mice (if available)

Note: CatSper-deficient mice [CatSper1- or CatSperd-null mice (Ren et al., 2001; Chung et al., 2011)] are used as a negative control to support the proof-of-concept in the study. If the lines are not applicable, the negative control can be replaced by pharmacological treatment using a CatSper inhibitor, such as NNC 05-0396.

• Nunc™ 4-Well Dishes for IVF (Thermo Fisher Scientific™, 144444)
• Hemocytometer
• A 1.5-mL microcentrifuge tube
• A 15-mL centrifuge tube
• CellVision 4 Chamber 20 micron (CellVision, CV 1020-4CH)
• Phosphate-buffered saline (Sigma-Aldrich, P4417)
• A measure of 0.4% HCl in distilled water (J.T. Baker, 9535-01)
• EmbryoMax^®^ M2 Medium (1X), Liquid, with Phenol Red (EMD Millipore, MR-015-D)
• EmbryoMax^®^ Human Tubal Fluid (HTF) (1X), Liquid, for Mouse IVF (EMD Millipore, MR-070-D)
• Modified HEPES-buffered HTF, H-HTF, pH 7.4 (Chung et al., 2017; Hwang et al., 2019)
  − 92 mM NaCl (AmericanBio, AB01915)
  − 4.7 mM KCl (J.T.Baker, 3040-01)
  − 0.2 mM MgCl_2_ (J.T.Baker, 2444-01)
  − 0.37 mM KH_2_PO_4_ (J.T.Baker, 3246-01)
  − 2.78 mM dextrose (J.T.Baker, 1920-01)
  − 0.33 mM sodium pyruvate (Sigma-Aldrich, P5280)
  − 18.3 mM sodium lactate (J.T.Baker, V034-08)
  − 10 mM HEPES (AmericanBio, AB00892)
• Dimethyl sulfoxide, DMSO (AmericanBio, AB03091)
• Pluronic™ F127, 20% solution in DMSO (Invitrogen, P3000MP)
• 20 mM stock of BAPTA-AM (Calbiochem, 196419) in DMSO
• Distilled water (Ultrapure grade)
• 5 mM stock of NNC 55-0396 hydrate (Sigma-Aldrich, N0287) in distilled water

### Equipment and instruments

• Microcentrifuge
• CO_2_ incubator
• Computer-assisted semen analysis system

Note: *The protocol described here is based on the SCA CASA* ^*®*^ *system.*

 - Inverted microscope (Nikon E200)
 - CMOS camera (Basler AG, acA1300-200 μm)
 - Warm stage
 - SCA^®^ CASA system analyzer (MICROPTIC)
 • Slide warmer (C&A Scientific Co., Inc.)

## Methods

### Basic principles and the overall procedure

The current protocol has been established to examine normal CatSper activity by analyzing motility changes depending on [Ca^2^]_i_ (Hwang et al., 2019). Sperm lose their motility by intracellular Ca^2+^ chelation (Marquez et al., 2007); CatSper is the main Ca^2+^ channel that introduces extracellular Ca^2+^ into the mammalian sperm. Thus, the previous study (Hwang et al., 2019) hypothesized the following:

Impaired sperm motility by intracellular Ca^2+^ chelation could be recovered if [Ca^2+^]_i_ is restored by the normal function of the CatSper channel.

**FIGURE 1:**
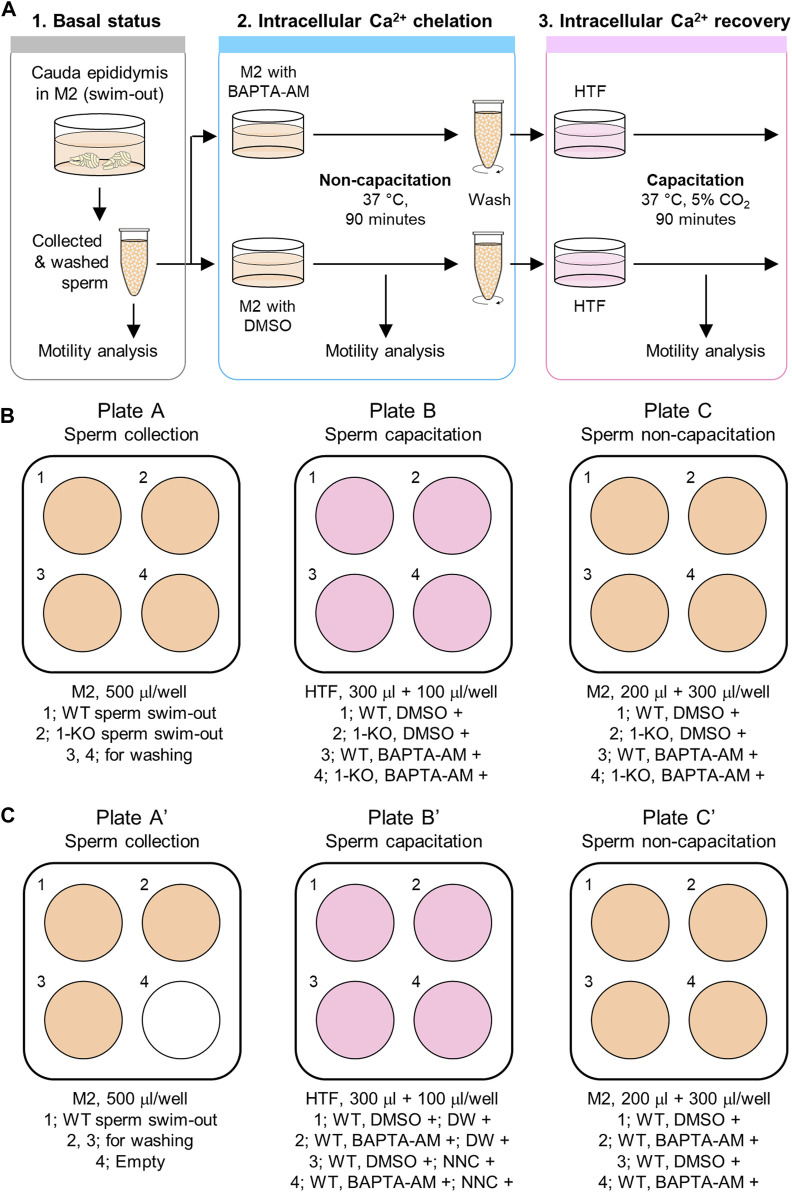
Procedure of the sperm Ca^2+^-handling assay. **(A)** Ca^2+^-handling assay to analyze [Ca^2+^]_i_-dependent sperm motility changes. Sperm motility is analyzed under three different conditions. First, the motility of fresh epididymal sperm is examined after swim-out (1. Basal status). Next, the sperm are incubated in a non-capacitating medium, M2, supplemented with an intracellular Ca^2+^ chelator, BAPTA-AM (2. Intracellular Ca^2+^ chelation status). Last, after incubation with BAPTA-AM for intracellular Ca^2+^ chelation, the sperm are washed and incubated in a capacitating medium, HTF, to activate the CatSper channel for extracellular Ca^2+^ influx into the sperm (3. Intracellular Ca^2+^ recovery status). Sperm motility is analyzed for 90 min of incubation in both the second and third steps. DMSO is used as a vehicle in this assay. **(B,C)** Media and plate preparation for the assay with the CatSper-deficient sperm **(B)** or pharmacological inhibition of the CatSper channel using NNC 05-0396 **(C)** for negative control. Shown are the 4-well plates and media information described in the Methods. WT, wild type; 1-KO, *CatSper1*-null; DW, distilled water; and NNC, NNC 05-0396.

This protocol compares the motility parameters of sperm depending on BAPTA-AM treatment for intracellular Ca^2+^ chelation. Thus, the sperm motility parameters are analyzed in three statuses, as described below (Figure 1A):

*Step 1*. Basal status: Non-capacitated, before inducing [Ca^2+^]_i_ chelation
*Step 2*. Intracellular Ca^2+^ chelation status: Non-capacitated, with BAPTA-AM
*Step 3*. Intracellular Ca^2+^ recovery status: Capacitation induced, without BAPTA-AM

First, the motility parameters of non-capacitated sperms are analyzed before BAPTA-AM treatment to chelate the intracellular Ca^2+^ (step 1). Next, sperm motility is examined during intracellular Ca^2+^ chelation under the non-capacitating condition supplemented with BAPTA-AM (step 2). Since sperm motility is altered following the removal of free intracellular Ca^2+^, the sperm will gradually lose motility during intracellular Ca^2+^ chelation by BAPTA-AM. Once the [Ca^2+^]_i_ is buffered at a marginal level, a severe reduction in the total sperm motility is seen. Following this, the sperm is washed, capacitation is induced without BAPTA-AM, and motility changes are analyzed (step 3). If CatSper is functioning normally, the channel is activated during capacitation and extracellular Ca^2+^ is introduced into the sperm. This will elevate [Ca^2+^]_i_ in the sperm and enable it to develop hyperactivated motility as well as recover motility. If CatSper is functioning abnormally, such as CatSper-deficient sperm, the sperm will not be able to recover motility and will fail to develop hyperactivated motility. Thus, the functional normality of the CatSper channel can be evaluated by analyzing motility changes during intracellular Ca^2+^ chelation and restoration.

## Before the start

The procedure to prepare the required reagents described in this study is basically for experiments that compare the motility changes of sperm from two animals (WT and CatSper1-null males). An experimental procedure using 1 animal (WT) with the pharmacological treatment of a CatSper inhibitor, NNC 05-0396, is also described— !) for negative control with pharmacological treatment—as an alternative negative control instead of the CatSper1-null sperm. Thus, the number of tubes, wells in plates, and/or the amount of the medium will need to be adjusted according to the number of animals being used in the experiment.

1. All wells of a 4-well plate (plate A; Figure 1B) are filled with 500 μL of M2 medium and kept warm at 37 °C.

!) For negative control with pharmacological treatment, three wells of a 4-well plate (plate A’; Figure 1C) are filled with 500 μL of M2 medium.

2. All wells of another 4-well plate are filled with 300 μL of HTF medium (plate B; Figure 1B), and two microcentrifuge tubes are prepared with 1.5 mL of HTF medium. The HTF medium in the 4-well plate and the tube (cap opened) need to be pre-incubated at 5% CO_2_, 37 °C humidifying environment for at least 1 h.

!) For negative control with pharmacological treatment, 0.4 μL of distilled water or the same volume of 5 mM NNC 05-0396 stock are added to two wells per each (plate B’; Figure 1C).

3. 5 mL of modified H-HTF medium is prepared in a 15-mL centrifugal tube and kept warm at 37 °C.
4. 2 mL of M2 medium is prepared in a centrifugal tube and kept warm at 37 °C.
5. 190 μL of 0.4% HCl solution is added in a microcentrifuge tube.
6. Two microcentrifuge tubes are prepared with 500 μL of PBS.
7. 2 μL of 20 mM of BAPTA-AM is mixed in DMSO and 2 μL of Pluronic F-127 (F-127). Also, DMSO and F-127 are mixed to the same ratio for control (vehicle).

Note: F-127 is required to disperse the esterification of the acetoxymethyl (AM) group in BAPTA-AM, which will cage the Ca^2+^ chelator in the treated cell. Thus, BAPTA-AM treatment without F-127 is functional.

Note: BAPTA-AM stock should be preserved at −20 °C until use; the stock should be mixed with F-127 just before euthanizing the mice.

Note: F-127 in DMSO is sticky at room temperature (RT). Thus, it should be collected slowly from the tube.

### Collection of epididymal sperm

1. Mice are euthanized in accordance with the guidelines approved by the Institutional Animal Care and Use Committee (IACUC).
2. Two cauda epididymides are taken out from a euthanized mouse, and the tissues are gently squeezed to remove blood. The collected tissues are placed in PBS in the microcentrifuge tubes at RT.
3. The tissues are moved to the M2 medium in plate A. Both epididymides from the same animal are placed into one well and cut 2–3 times using scissors followed by incubation on a 37°C warm plate for 10 min to let the sperm swim out of the epididymis.

Note: The M2 medium is buffered with HEPES. Thus, the medium should not be incubated in a CO_2_ incubator as this will alter its pH.

4. A new 4-well plate is prepared with 200 μL of pre-warmed M2 medium, and 0.25 μL of BAPTA-AM/F-127 or DMSO/F-127 mixture (plate C and C’; Figures 1B, C, respectively) is added and mixed well by gently pipetting.

Note: The sperm are placed in each well of these plates (plate C and C’; Figures 1B, C, respectively) in a 3.5 × 10^6^ cells/mL concentration. As the final volume in the wells will be 500 μL, the concentrations of BAPTA-AM and DMSO will be 5 μM and 0.05% (v/v), respectively. The F-127 concentration will be 0.01% (w/v).

5. The M2 medium with sperm is transferred to new microcentrifuge tubes using a micropipette. The tubes are placed upright for 5 min so that any non-sperm cells and debris can sediment.

Note: All pipetting for the sperm should be performed gently to minimize any physical damage to the cells.

6. 400 μL of the sperm in the M2 medium are gently taken from the top and transferred to a new microcentrifuge tube followed by centrifugation at 500 *g* for 3 min at RT.
7. The supernatant is discarded, and the cells are resuspended with 300 μL of pre-warmed M2 medium at 37 °C.

Note: The sperm cells are sedimented rather than pelleted tightly.

8. 10 μL of the sperm in the M2 medium are taken and mixed with 190 μL of 0.4% HCl in a microcentrifuge tube by gentle tapping; 6–8 μL of the mixture is placed in the hemocytometer to count the sperm number and calculate the concentration.
9. A small volume of the sperm in the M2 medium is taken and mixed with the pre-warmed M2 medium to prepare 50 μL of sperm in the M2 medium with a concentration of 3.5 × 10^6^ cells/mL. The sperm in this small volume of M2 are used to record their motility before intracellular Ca^2+^ chelation with BAPTA-AM.
10. 1.75 × 10^6^ cells in the M2 medium are added to the wells containing BAPTA-AM or DMSO in plate C (3.5 × 10^6^ cells/mL) and filled up to 500 μL with the pre-warmed M2 medium. The 4-well plate (plate C) is placed on the slide warmer, and the sperm cells are incubated at 37 °C for 90 min to analyze their motility changes using CASA.
!) For negative control with pharmacological treatment, 1.75 × 10^6^ cells in the M2 medium are added to all wells containing either BAPTA-AM or DMSO (plate C’; Figure 1C). Later, sperm in two wells under the same condition are capacitated with or without NNC 05-0396.

### Motility analyses using the CASA system

1. The SCA CASA system, microscope, and slide warmer equipped with a microscope are turned on. The temperature of the slide warmer should be maintained at 37 *°*C.
  Note: As the operation of CASA systems may be different depending on the brands and companies, a detailed configuration and protocol on how to use the software are not described here.

Note: Sperm motility should be measured on the slide warmer equipped with a microscope, which is set to 37 °C, throughout the experiment.

**FIGURE 2:**
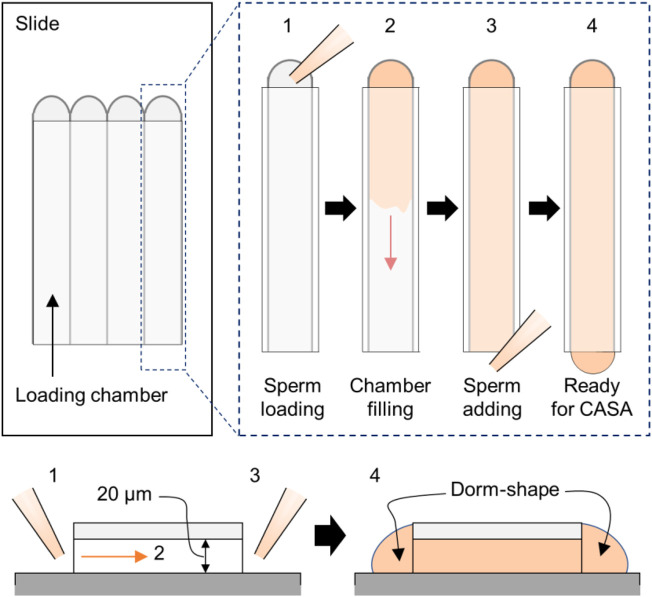
CASA application in the Ca^2+^-handling assay. The cartoon shows sample loading in the CASA imaging chamber. Shown are the top–down (*top*) and side (*bottom*) views. In brief, the sperm-containing medium is loaded first into one side (step 1), and we wait until the solution spreads thoroughly to the end of the other side (step 2). Next, the remaining sperm solution is added to the open-end side to create a dome shape on each end to prevent evaporation (step 3). Then, sperm in the chambers are ready to be analyzed (step 4).

Notes: The time points to measure the motility parameters could be changed according to the experiment.

Note: Each experiment carried out to analyze sperm motility will take some time. Therefore, the incubation of sperm from different animals might be initiated with 5–10-min intervals.

Note: In general, the curvilinear velocity (VCL) of WT sperm will drop significantly and be lowered to the basal level 15–30 min following incubation with 5–10 µM of BAPTA-AM. If the sperm’s swimming speed is not altered rapidly, it could be due to the activity loss of BAPTA-AM.

2. 20 μL of the sperm-containing M2 medium (non-capacitated) is taken, which does not carry either vehicle (DMSO) or BAPTA-AM, and placed in the chamber.
Note: The sperm-containing medium is loaded from one side. Once the solution fills up the chamber, extra solution is placed on each side of the path to prevent evaporation during the experiment, as depicted in Figure 2.
3. The chamber is placed on the warm stage equipped with the microscope, and sperm motility parameters are analyzed using the CASA system. Usually, over 200 total sperms need to be measured per experiment.
4. The recorded information is saved, and a new experiment tab is opened in the software.
5. Steps 2–4 are repeated to measure sperm motility after 15, 30, and 90 min of incubation in an M2 medium supplemented with DMSO or BAPTA-AM.

6. After 90 min of incubation, the remaining sperm (approximately 400 μL) are transferred into microcentrifuge tubes and centrifuged at 700 *g* for 2 min.
!) For negative control with pharmacological treatment, the sperm incubated under the same condition (approximately 900 μL) are transferred to one tube and centrifuged under the same condition as above.
7. The supernatant is removed, and the sperm are pelleted with 800 μL of H-HTF medium followed by another centrifugation at 700 *g* for 2 min to wash out the vehicle and BAPTA-AM in the media. This step is repeated one more time.

Note: A modified H-HTF medium lacking HCO^3^- and Ca^2+^ is used in this study, as per the original publication (Hwang et al., 2019). However, a normal H-HTF medium could be also applicable which does not affect the results dramatically (Hwang et al., 2022).

8. The supernatant is removed, and the sperm pellet is resuspended with 300 μL of pre-incubated HTF medium and centrifuged at 700 *g* for 2 min.
9. The supernatant is removed, and sperm pellets are resuspended with 100 μL of HTF pre-incubated in a microcentrifuge tube. The sperm elute is transferred to each well of plate B (Figure 1B). Incubation is started at 5% CO_2_, 37 *°*C condition to induce capacitation.
!) For negative control with pharmacological treatment, the sperm pellet is eluted in each tube with 200 μL of the HTF media in a microcentrifuge tube and 100 μL of the elute is transferred to the HTF medium supplemented with distilled water or NNC 05-0396 in a 4-well plate. The final volume in each well is 400 μL, and the concentration of NNC 05-0396 is 5 μM.
10. The sperm motility is measured at 15, 60, and 90 min after inducing capacitation using the CASA system.

Note: *These time points can be changed according to the experiment.*

11. The results are exported, and the motility changes are compared statistically.

## Anticipated results and discussion

The expected results are described based on the previous study (Hwang et al., 2019).

### Lowering [Ca^2+^]_i_ impairs sperm motility

**FIGURE 3:**
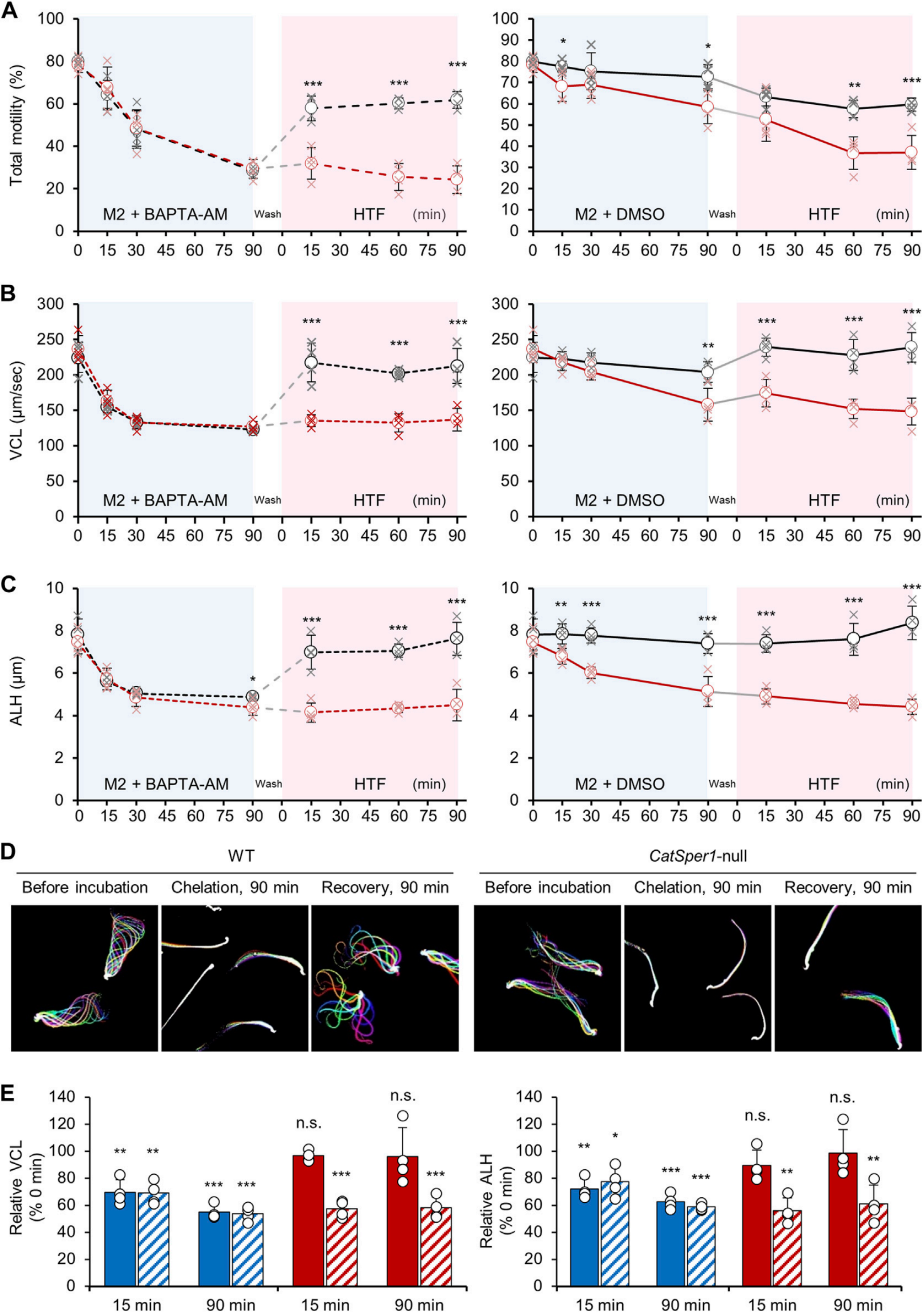
CatSper-dependent sperm motility changes by handling [Ca^2+^]_i_. **(A–C)** Changes in the total sperm motility **(A)**, curvilinear motility [VCL, **(B)**], and amplitude of head displacement [ALH, **(C)**] during the Ca^2+^-handling assay. Shown are the changes in the motility parameters of WT (black, N = 4) and *CatSper1*-null (red, N = 4) sperm by chelating intracellular Ca^2+^ followed by inducing capacitation (*left*). DMSO is used as the vehicle (*right*). The data are represented by the mean ± SD (N = 4 for each strain). Cross marks at each time point represent values for sperm motility parameters from individual males. Statistical comparisons are performed between the WT and *CatSper1*-null sperm at each time point using Student’s *t*-test. **(D)** Images for the flagellar waveform changes of the sperm from WT (*left*) and *CatSper1*-null (*right*) males during the Ca^2+^-handling assay. Flagellar waveforms of head-tethered WT and *CatSper1*-null sperm are imaged before (each *left*) and after 90 min (each *middle*) incubation with BAPTA-AM and 90 min recovery in the HTF medium after washing (each *right*). Shown are merged images taken for two beating cycles. **(E)** Relative changes in the VCL (*left*) and ALH (*righ*t) of the WT (filled) and *CatSper1*-null (sashed) sperm in the Ca^2+^-handling assay. Shown are the relative changes after intracellular Ca^2+^ chelation (blue bars) and inducing capacitation (red bars). The values at each time point are normalized to those at 0 min and statically compared using Student’s *t*-test. The data are represented as the mean ± SD (N = 4 for each strain). Circles indicate the relative changes in the motility parameters in the sperm from each male. *p < 0.05; **p < 0.01; and ***p < 0.001. n.s., non-significant. The charts and flagellar waveform images are reproduced from a previous study (Hwang et al., 2019) with the permission of *Cell*.

As depicted in a previous study (Marquez et al., 2007), treatment with the intracellular Ca^2+^ chelator, BAPTA-AM, dramatically reduces total sperm motility and severely alters the motility parameters (Figure 3). The total motility of both WT and *CatSper1*-null sperm was severely reduced following a 90-min incubation with 5-µM BAPTA-AM under the non-capacitating condition (Figure 3A, left; WT, 79.83% ± 3.29% to 28.36% ± 0.93%; *CatSper1*-null, 78.2% ± 2.09% to 29.3% ± 2.09%). In addition, the motility parameters, VCL, and amplitude of the lateral head (ALH), were also severely reduced after 90 min of incubation with BAPTA-AM in the sperm from both WT (VCL, 224.57 ± 12.22 μm/s to 123.03 ± 1.43 μm/s; ALH, 7.82 ± 0.42 µm to 4.87 ± 0.05 µm) and *CatSper1*-null (VCL, 236.46 ± 10.83 μm/s to 126.71 ± 4.05 μm/s; ALH, 7.45 ± 0.31 µm to 4.38 ± 0.21 µm) mice (Figures 3B, C, both left). Notably, despite the gradual decrease in the total motility for 90 min, the VCL and ALH values dropped more rapidly, and 15–30 min of incubation with BAPTA-AM is enough to lower these motility parameters to the basal level (Figures 3A–C). These results clearly demonstrate that lowering [Ca^2+^]_i_ by BAPTA-AM rapidly alters the overall sperm swimming ability. Interestingly, despite the absence of BAPTA-AM, *CatSper1*-null sperm motility was also reduced after 90 min of incubation under the non-capacitating condition (Figure 3A, *right*; 78.25% ± 2.09% to 58.49% ± 4.58%). In addition, the VCL and ALH values were also severely decreased after 90 min of incubation without BAPTA-AM (Figures 3B, C, *right* each; VCL, 236.46 ± 10.83 μm/s to 157.96 ± 13.49 μm/s; ALH, 7.45 ± 0.31 µm to 5.13 ± 0.41 µm). These results are consistent with previous studies that analyzed the motility changes of *CatSper1-, 2*-, *3*-, and 4-null sperm (Qi et al., 2007) or *CatSperd*-null sperm (Hwang et al., 2022) under non-capacitating conditions. One possibility could be that the intracellular Ca^2+^ is rapidly depleted and failed to maintain proper [Ca^2+^]_i_ in the CatSper-deficient sperm. CatSper could marginally introduce extracellular Ca^2+^ into the sperm under non-capacitating and capacitating conditions (Kirichok et al., 2006; Hwang et al., 2019). Thus, the CatSper-deficient sperm may keep consuming and/or extruding free intracellular Ca^2+^ but fail to recharge it, which will eventually impair sperm motility even under the non-capacitating condition without BAPTA-AM.

### [Ca^2+^]_i_-lowered sperm recover motility in a CatSper-dependent manner during capacitation

Both the WT and *CatSper1*-null sperm were treated with BAPTA-AM for 90 min followed by washing with a modified H-HTF medium to terminate any additional BAPTA caging in the sperm. The washed sperm were further incubated under the capacitating condition for 90 min. Although the WT sperm could successfully rescue their motility, the *CatSper1*-null sperm failed to do this for 90 min of capacitation. The total motility of the WT sperm increased rapidly (Figure 3A, left) after inducing capacitation for 15 min (28.36% ± 0.93% to 57.80% ± 3.35%), and the value was maintained for 90 min of capacitation (61.78% ± 2.31%). In contrast, the total motility of the *CatSper1*-null sperm did not significantly increase after washing and inducing capacitation for 90 min (29.34% ± 2.55% to 31.83% ± 4.32%). In addition, the VCL (Figure 3B, left) and ALH (Figure 3C, left) values in the WT sperm increased dramatically after only 15 min of capacitation (VCL, 123.03 ± 1.43 μm/s to 217.37 ± 15.81 μm/s; ALH, 4.87 ± 0.05 µm to 6.98 ± 0.46 µm) and were maintained or further increased after 90 min of capacitation (VCL, 212.59 ± 14.23 μm/s; ALH, 7.62 ± 0.44 µm). Furthermore, the WT sperm recovered from lowered [Ca^2+^]_i_ can successfully develop hyperactivated motility (Figure 3D; Hwang et al., 2019). However, the motility parameters were not rescued in the *CatSper1*-null sperm following 90 min of capacitation (VCL, 126.71 ± 4.05 μm/s to 136.84 ± 9.13 μm/s; ALH, 4.38 ± 0.21 µm to 4.51 ± 0.43 µm). The WT sperm capacitated with NNC 055-0396 failed to recover the motility parameters after intracellular Ca^2+^ chelation (Supplementary Figure S1) like the CatSper-deficient sperm. All the results clearly demonstrate that sperm motility parameters are recovered in a CatSper-dependent manner after intracellular Ca^2+^ chelation. The CatSper-mediated recovery of sperm motility is also supported by a relative comparison of the motility parameters before and after inducing capacitation (Figure 3D). Notably, chelating intracellular Ca^2+^ for 90 min seems to barely affect the recovery process due to the potential introduction of excessive BAPTA-AM. Recovery statuses by inducing capacitation are not significantly different within the groups incubated with BAPTA-AM for 30 or 90 min, which lowers the motility parameters to the basal level (Supplementary Figure S2).

Previous studies demonstrated that VCL and ALH values were recovered in the *Efcab9*-null sperm but not in the *CatSpert*-null sperm, in which the CatSper current is at ∼50% and 10% level compared to the current in the WT sperm, respectively (Hwang et al., 2019; Hwang et al., 2022). These results indicate that the Ca^2+^-handling assay is applicable to detect the altered CatSper function in which the current is lower than 50% of the normal ranges despite its lesser sensitivity than patch clamping.

The total motility and motility parameters in the CatSper-deficient sperm could transiently increase after washing (Hwang et al., 2019; Hwang et al., 2022). Originally, the *CatSper1*-or *CatSperd*-null sperm were expected to fail to recover their motility parameters after washing and inducing capacitation due to the absence of CatSper. However, the motility parameters were marginally recovered in the CatSper-deficient sperm after washing (refer to the 15-min point after inducing capacitation in Figures 3A, B). Presumably, this might be due to the transient increase of free Ca^2+^ released from the storage organelle (e.g., mitochondria) during the washing step, which needs to be further examined.

Although the marginal motility rescue was observed shortly, total motility and the parameters in CatSper-deficient sperm are eventually compromised during capacitation. The above results clearly demonstrate that sperm motility is recovered by restoring [Ca^2+^]_i_ via CatSper-mediated Ca^2+^ entry during capacitation.

This new method can examine the functional normality of the CatSper channel by analyzing sperm motility changes. Although this method is currently validated in mouse sperm, optimizing this method in sperm from other species would contribute to expanding the methodology to analyze CatSper function. There is a possibility that altered upstream pathways also affect abnormal CatSper function in sperm. Thus, direct methods to open CatSper specifically, such as intracellular alkalinization using NH_4_Cl, could be applicable in this protocol to distinguish altered CatSper function by upstream signaling pathways. Those modifications remain to be further tested, and updated protocols will also contribute to examining the functional normality of the CatSper channel in mammalian sperm.

## Data Availability

The data analyzed in this study are subject to the following licenses/restrictions: raw CASA data are from a previous study (Hwang et al., 2019; Cell) and this study. Requests to access these datasets should be directed to JH, jyhwang@pusan.ac.kr.

## Supplementary material

SUPPLEMENTARY FIGURE S1 Motility changes of WT sperm capacitated with or without NNC 05-0396 (NNC) after intracellular Ca2+ chelation. Shown are the changes in total motility **(A)**, curvilinear velocity [VCL, **(B)**], and amplitude of head displacement [ALH, **(C)**] of WT sperm capacitated with 5 μM of NNC 05-0396, a CatSper inhibitor (NNC +, dashed lines). Distilled water was used for control (control, solid lines). Impaired motility changes by NNC 05-0396 were examined in the capacitating sperm, which were incubated in the M2 medium supplemented with BAPTA-AM (left, red lines) or DMSO (right, black lines) The data are represented by the mean ± SD (N=3). Triangles and cross marks represent the values for the motility parameters from individual males, whose sperm were capacitated with or without NNC 05-0396, respectively. Statistical comparisons are performed within control and NNC-treated groups at each time point using Student’s *t*-test. *p<0.05; **<0.01; and ***<0.001.

SUPPLEMENTARY FIGURE S2 Effects of incubation time for intracellular Ca2+ chelation to rescue motility parameters. **(A)** Changes of curvilinear motility (VCL, top) and amplitude of head displacement (ALH, bottom) values after incubation with BAPTA-AM for 30 (left) or 90 (right) minutes. Shown are the changes in VCL and ALH values incubated with an M2 medium supplemented with BAPTA-AM (red) or DMSO (black). Statistical comparisons are conducted within groups incubated with DMSO or BAPTA-AM using Student’s *t*-test (N=3). *p<0.05; **p<0.01; and ***P<0.001. **(B)** Relative comparison of VCL (top) and ALH (bottom) values in the capacitating (Cap) sperm after chelating intracellular Ca2+ for 30 or 90 minutes. Shown are the relative VCL and ALH values of sperm capacitated for 15, 60, and 90 minutes after intracellular Ca2+ chelation. The values are normalized by those from sperm incubated with DMSO at the same time points. None of the relative values between 30 and 90 minutes of intracellular Ca2+ chelation are significantly different at each time point (N=3, each). Statistical comparisons are performed with Student’s *t*-test. The data are represented by the mean ± SD. Values from individual animals are marked in crosses **(A)** and circles **(B)** at each time point. Data for the 90-minute intracellular Ca2+ chelation are from those shown in Supplementary Figure 1.
