# Supplementary figures and images for: Analysis of Ca2+-mediated sperm motility to evaluate the functional normality of the sperm-specific Ca2+ channel, CatSper
Source: Front Cell Dev Biol. 2024 Feb 7;12:1284988. doi: 10.3389/fcell.2024.1284988 (PMC10879342; doi:10.3389/fcell.2024.1284988)

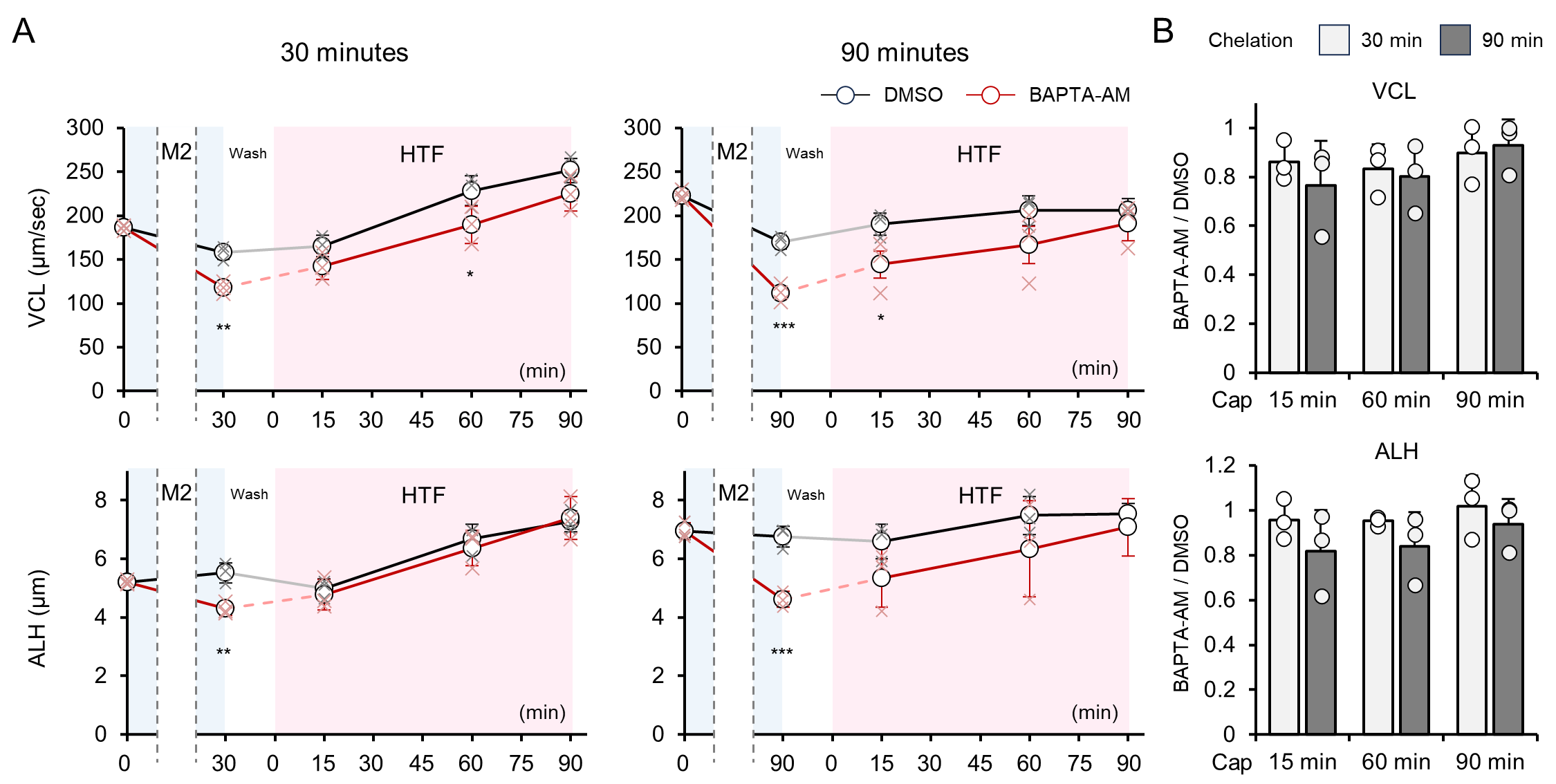

Supplement: Supplementary file 1 [file Image2.tif]

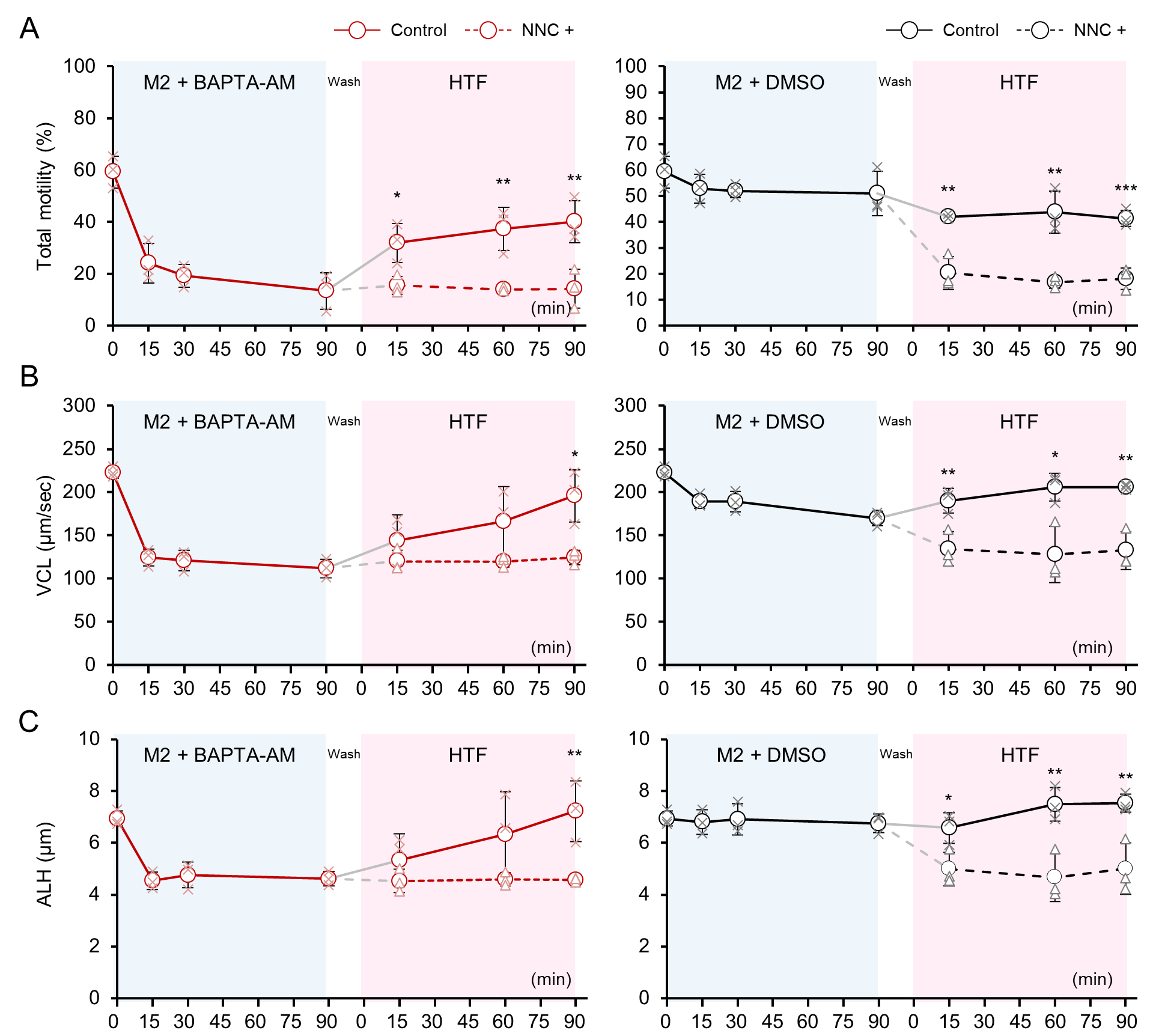

Supplement: Supplementary file 2 [file Image1.tif]
